# Reviewer acknowledgement 2015

**DOI:** 10.1186/s12941-016-0160-2

**Published:** 2016-08-01

**Authors:** Hakan Leblebicioglu

**Affiliations:** Ondokuz Mayis University Medical School, Samsun, Turkey

## Contributing reviewers

*Annals of Clinical Microbiology and Antimicrobials* would like to thank the following colleagues for their assistance with peer review of manuscripts for the journal in 2015.

**Hossam Abdallah**

USA

**Ramin Abiri**

Iran

**Alfredo Aires**

Portugal

**Mohamed Al-Agamy**

Saudi Arabia

**Cristiano Alicino**

Italy

**Nuno Almeida**

Portugal

**Mustafa Altindis**

Turkey

**Maria Alves**

Portugal

**Mickael Aoun**

Belgium

**Jesus Aranda**

Spain

**Ferhat Arslan**

Turkey

**Takanori Asakura**

South Korea

**Hossam Ashour**

USA

**Sébastien Bailly**

France

**Ayse Batirel**

Turkey

**Michael A. Benusic**

USA

**Anna Berthold-Pluta**

Poland

**Douglas Biedenbach**

USA

**Raja Biswas**

USA

**Hurrem Bodur**

Turkey

**Daniel Boyle**

USA

**Bülent Bozdogan**

Turkey

**Dragana Bozic**

Serbia

**Anne Karin Brigtsen**

Norway

**Zahid Butt**

USA

**Luisfa Camargo**

Brazil

**Emmanuelle Cambau**

France

**Gerald Capraro**

USA

**Jean Carlet**

France

**Ana Paula D’Alincourt Carvalho-Assef**

Brazil

**Antonella Castagna**

Italy

**Nevreste Celikbilek**

USA

**Sheng Chen**

Hong Kong

**Yen-Hsu Chen**

Taiwan

**Chien Hung Chen**

Taiwan

**Cheng-Hsun Chiu**

Taiwan

**Chishih Chu**

Taiwan

**Rungtip Chuanchuen**

Thailand

**Ihsan Hakki Ciftci**

Turkey

**Serap Cosansu**

Turkey

**Jared Crandon**

USA

**Burke Cunha**

USA

**Lidia Dalfino**

Italy

**Ziad Daoud**

Lebanon

**Vincenzo De Francesco**

Italy

**Tuna Demirdal**

Turkey

**Venkata Siva Charan Devanaboyina**

USA

**Laurent Dortet**

France

**Robert Doyle**

USA

**Elif Doyuk**

USA

**Kwabena O. Duedu**

Ghana

**Percin Duygu**

Turkey

**Bart Eijkelkamp**

Australia

**Mohamed El Zowalaty**

Qatar

**Nazif Elaldi**

Turkey

**Halil Er**

Turkey

**Onder Ergonul**

Turkey

**Unal Erkorkmaz**

Turkey

**Saban Esen**

Turkey

**Marco Falcone**

Italy

**Liliana Fernandez-Canigia**

Argentina

**Thomas Fletcher**

UK

**Julia Garcia-Diaz**

USA

**Serap Gencer**

Turkey

**Montserrat Gimenez**

Spain

**Ivana Goic-Barisic**

Croatia

**Hasan Tahsin Gözdaş**

Turkey

**Ezekiel Green**

South Africa

**Ertugrul Guclu**

Turkey

**Hüseyin Güdücüoğlu**

USA

**Selami Gunal**

Turkey

**Saban Gürcan**

Turkey

**Muhammad Nazrul Hakim**

Malaysia

**Valeria Haley**

USA

**Katherine Hammer**

Australia

**Dalal Hammoudi**

Lebanon

**Rodrigo Hasbun**

USA

**Karl Hassan**

Australia

**Alice Hayford**

USA

**Miriam Hernandez-Porto**

Spain

**Salih Hosoglu**

Turkey

**Ana Hoxha**

USA

**Moises Huaman**

USA

**Nitaya Indrawattana**

Thailand

**Salmah Ismail**

Malaysia

**Kenta Ito**

Japan

**Kausar Jabeen**

USA

**George Jacoby**

USA

**Anders Jensen**

Denmark

**Seok Hoon Jeong**

South Korea

**Oguz Karabay**

Turkey

**Drosos Karageorgopoulos**

Greece

**Zahra Kassamali**

USA

**Solen Kerneis**

France

**Byung-Wook Kim**

South Korea

**Yonghyun Kim**

USA

**Michelle Kip**

The Netherlands

**Teruo Kirikae**

Japan

**Leif Kirsebom**

Sweden

**Milan Kojic**

Serbia

**Mehmet Koroglu**

Turkey

**Ozgen Koseoglu Eser**

Turkey

**Esther Kuenzli**

Switzerland

**Ravina Kullar**

USA

**Duangkamol Kunthalert**

Thailand

**Olga Lage**

Portugal

**Antonietta Lambiase**

Italy

**Dortet Laurent**

France

**David Lebeaux**

France

**Jae Gil Lee**

South Korea

**Wen Sen Lee**

Taiwan

**A. Lerner**

Israel

**Mingchun Li**

China

**Yu Li**

USA

**Kung-Hao Liang**

Taiwan

**Adamantia Liapikou**

Greece

**Peter Lipke**

USA

**Lin Liu**

China

**Jh Liu**

China

**Qi Liu**

USA

**Ethel Maciel**

Brazil

**Colin Rae Mackenzie**

Germany

**Caterina Mammina**

Italy

**Mohammed Abul Manchur**

Bangladesh

**Santi Mandal**

USA

**Manel Marzouk**

USA

**Sandra Mccurdy**

USA

**Shaheen Mehtar**

South Africa

**Kristina Mena**

USA

**Werner Mendling**

Germany

**Maia Merabishvili**

Belgium

**Gokhan Metan**

Turkey

**Virginie Mick**

France

**Egidia Miftode**

UK

**Morteza Milani**

USA

**Michael Moen**

USA

**Tor Monsen**

Sweden

**Billy Ngasala**

Sweden

**David Nicolau**

USA

**Saad Nseir**

France

**Nadia Oulahal**

France

**Resar Ozaras**

Turkey

**Resit Ozkanca**

Turkey

**Rabindra Padhy**

India

**Helene Pailhories**

France

**Timothy Palzkill**

USA

**Costas C. Papagiannitsis**

USA

**Joseph Papaparaskevas**

Greece

**Ema Paulovicova**

Slovakia

**Julie Paulsen**

Norway

**Avi Peretz**

Israel

**Rutger Persson**

Switzerland

**Mathieu Picardeau**

France

**Piotr Pierzchalski**

Poland

**Monika Pobiega**

USA

**István Pócsi**

Hungary

**Laurent Poirel**

Switzerland

**Virginia Pomar**

Spain

**Alfredo Ponce-De-Leon**

USA

**Anna Psaroulaki**

Greece

**Rayane Rafei**

USA

**Chinnarajan Ravindran**

USA

**Mehdi Razzaghi-Abyaneh**

USA

**Sujan Reddy**

USA

**Marilyn Roberts**

USA

**Daniela Ramos Rodrigues**

Brazil

**Camilla Rodrigues**

India

**Leandro Reus Rodrigues Perez**

USA

**Sabrina Royer**

Brazil

**Federico Ruiz**

Spain

**Maja Rupnik**

Slovenia

**Kazim Sahin**

Turkey

**Gyan Sahukhal**

USA

**Yasra Sarwar**

Pakistan

**Francesco Sbrana**

Italy

**Frieder Schaumburg**

Germany

**Caroline Schneeberger**

The Netherlands

**John Osei Sekyere**

USA

**Samy Selim**

Saudi Arabia

**Bilge Sener**

Turkey

**Sunil Shaunak**

UK

**Suchitra Shenoy**

India

**Naseh Sigari**

Iran

**Hanna Sikorska**

Canada

**Jesús Silva**

Mexico

**Oguz Resat Sipahi**

Turkey

**Younes Smani**

Spain

**Ali Somily**

Saudi Arabia

**Ellen Stobberingh**

The Netherlands

**Uwe Stroeher**

Australia

**Philip Suffys**

Brazil

**Sistla Sujatha**

USA

**Vidya Sundareshan**

USA

**Piotr Szweda**

Poland

**Morovat Taherikalani**

Iran

**Jeanette Teo**

Singapore

**Joshua Thaden**

USA

**Selma Tobudic**

Austria

**Fevzi Topal**

Turkey

**Bryan Troxell**

USA

**Athanassios Tsakris**

Greece

**Ephraim Tsalik**

USA

**Vedat Turhan**

Turkey

**Ediz Tütüncü**

Turkey

**Nurver Ulger**

Turkey

**Renáta Vadkertiová**

Slovakia

**Ali Vahidnia**

The Netherlands

**Monique Van Hoek**

USA

**Alkiviadis Vatopoulos**

Greece

**Matthew Wand**

UK

**Gilles Wandeler**

Switzerland

**Gamal Wareth**

Germany

**Henrik Westh**

Denmark

**Frank Wolschendorf**

USA

**Yonghong Xiao**

China

**Kimikazu Yakushijin**

Japan

**Keiko Yamada**

Japan

**Yoshio Yamaoka**

Japan

**Shinji Yamasaki**

Japan

**Keramettin Yanik**

Turkey

**Yoshihiko Yano**

Japan

**Mohamad Yasmin**

USA

**Chunmei Ying**

USA

**Heather Young**

USA

**Zerrin Yulugkural**

Turkey

**Heng Zheng**

China

**Paolo Zucca**

Italy

**Angelo Zullo**

Italy

